# FGF19 promotes epithelial-mesenchymal transition in hepatocellular carcinoma cells by modulating the GSK3β/β- catenin signaling cascade via FGFR4 activation

**DOI:** 10.18632/oncotarget.6185

**Published:** 2015-10-20

**Authors:** Huakan Zhao, Fenglin Lv, Guizhao Liang, Xiaobin Huang, Gang Wu, Wenfa Zhang, Le Yu, Lei Shi, Yong Teng

**Affiliations:** ^1^ School of Life Sciences and School of Bioengineering, Chongqing University, Chongqing, PR China; ^2^ Third Affiliated Hospital, Third Military Medical University, Chongqing, PR China

**Keywords:** FGF19, FGFR4, EMT, E-cadherin, GSK3β/β-catenin

## Abstract

Compelling evidence suggests that the epithelial-mesenchymal transition (EMT) correlates with aggressiveness of tumors and poor survival. FGF19 has been shown to be involved in EMT in cholangiocarcinoma and colorectal cancer, however, molecular mechanisms underlying FGF19-induced EMT process in hepatocellular carcinoma (HCC) remain largely unknown. Here, we show the expression of FGF19 is significantly elevated and negatively associated with the expression of E-cadherin in HCC tissues and cell lines. Ectopic FGF19 expression promotes EMT and invasion in epithelial-like HCC cells through repression of E-cadherin expression, whereas FGF19 knockdown enhances E-cadherin expression and hence diminishes EMT traits in mesenchymal-like HCC cells, suggesting FGF19 exerts its tumor progressing functions as an EMT inducer. Interestingly, depletion of FGF19 cannot abrogate EMT traits in the presence of GSK3β inhibitors. Furthermore, FGF19-induced EMT can be markedly attenuated when FGFR4 is knocked out. These observations clearly indicate that FGFR4/GSK3β/β-catenin axis may play a pivotal role in FGF19-induced EMT in HCC cells. As FGF19 and its specific receptor FGFR4 are frequently amplified in HCC cells, selective targeting this signaling node may lend insights into a potential effective therapeutic approach for blocking metastasis of HCC.

## INTRODUCTION

Hepatocellular carcinoma (HCC) is one of the most common malignancies and the third most leading cause of cancer associated mortality in the world [1]. Early metastasis is responsible for frequent relapse and high mortality of HCC and elucidation of the molecular mechanism underlying aggressive HCC is an urgent therapeutic need for suppression of metastasis [2, 3]. The epithelial-mesenchymal transition (EMT) of neoplastic hepatocytes has been considered to be a key event in metastasis and plays a critical role in progression of HCC [4]. The EMT process is governed by multiple molecular mechanisms and crucial steps, including the loss of E-cadherin function [5]. Low levels of E-cadherin have been observed in 58% of human primary HCC samples and are associated with a poor prognosis [6], demonstrating the pivotal role of this protein in cancer progression. Several repressor of E-cadherin, such as Snail1 and Twist, negatively regulate the expression of E-cadherin through binding to and repressing its promoter [7]. It has been shown that up-regulation of Snail1 and/or Twist was associated with down-regulation of E-cadherin [8].

Numerous studies indicate that the activation of Wnt/β-catenin signaling can promote transcriptional changes in order to drive EMT [9]. Deregulation of Wnt/β-catenin pathway has been associated with the development of HCC [10]. β-catenin levels are regulated by a multiprotein complex consisting of adenomatous polyposis coli (APC) tumor suppressor protein, axin, casein kinase 1 (CK1) and glycogen synthase kinase 3β (GSK3β) [11]. In steady-state conditions, cytoplasmic β-catenin is bound to this destruction complex and phosphorylated by GSK3β at specific serine/threonine residues located at the N-terminal region [12]. As a result, this sequential phosphorylation targets β-catenin for ubiquitination and ultimate degradation by the proteasomal pathway. In the aberrant conditions, inactivation of GSK3β by phosphorylation leads to β-catenin stabilization and accumulation in nucleus [13, 14]. Within the nucleus, β-catenin binds to lymphoid-enhancing factor/T-cell factor (LEF/TCF) and then initiates the transcription of its target genes, such as Snail1 and Twist [9].

Fibroblast growth factor 19 (FGF19) is a member of the hormone-like FGF family and has activity as an ileum-derived postprandial hormone [15, 16]. It shares high binding affinity with β-Klotho and together with the FGF receptor 4 (FGFR4), is predominantly targeted to the liver [17]. FGF15 is the rodent ortholog of human FGF19 from the views of their similar genomic structures and biological role [18]. It has been shown that FGF15/FGF19 induces the expression of the pro-fibrogenic and pro-tumorigenic connective tissue growth factor (CTGF) in hepatocytes [19]. In disease state, FGF19 is critical for the development and progression of a number of cancers, including HCC, breast cancer, prostate cancer and cholangiocarcinoma [20–23]. Recently, focal amplification frequency of FGF19 was identified in 12–14% of HCC clinical samples [20], which is positively correlated with tumor size, pathological stage and poor prognosis [24, 25]. FGF19 was also detected in cirrhosis, a preneoplastic condition that often leads to liver carcinoma [26]. FGF19 signaling involves activation of different intracellular pathways, including the mitogen activated protein kinase-extracellular signal-regulated kinase (MAPK-ERK) and β-catenin pathways [27]. More specifically, FGF19 increases GSK3β phosphorylation and activates β-catenin, leading to the activation of β-catenin/TCF4-regulated transcription in colon cancer cells [28].

Decrease in the tumorigenic and invasive capacity of colorectal cancer cells by FGFR4 silencing was accompanied by a decrease of the expression of Snail and Twist and an increase of E-cadherin expression [29]. In cholangiocarcinoma cells, E-cadherin expression had a lower expression, whereas N-cadherin, Snail1 and Vimentin had higher expressions under FGF19 stimulation, indicating that FGF19 can trigger the EMT process. However, this tendency fade away when FGFR4 was knocked down, suggesting FGFR4 is required for the FGF19-induced EMT in cholangiocarcinoma cells [23]. In HCC, treatment JHH7 cells with recombinant FGF19 protein leads to an increase in cell proliferation and invasion and a decrease in cell apoptosis [24]. In contrast, decreasing FGF19 and FGFR4 expression by siRNA significantly inhibits proliferation, invasion and increases apoptosis [24].

In this study, we investigated the events underlying the EMT of HCC cells. We have found for the first time that FGF19 and E-cadherin expressions are negatively correlated in HCC tissues and cell lines. Overexpression of FGF19 induces EMT traits through repression of E-cadherin and knocking it down produces an opposite effect. Furthermore, depletion of FGF19 cannot abrogate EMT traits in the treatment with GSK3β inhibitors. Inversely, FGF19-induced EMT can be markedly attenuated when FGFR4 is knocked out. These data provide a possible mechanism that FGF19/FGFR4/GSK3β/β-catenin/E-cadherin axis is largely responsible for the regulation of EMT in HCC cells. Targeting this signaling node therefore represents a potential therapeutic strategy for the treatment of HCC.

## RESULTS

### The expression levels of FGF19 and E-cadherin are negatively associated in HCC tissues and cell lines

**Figure 1:**
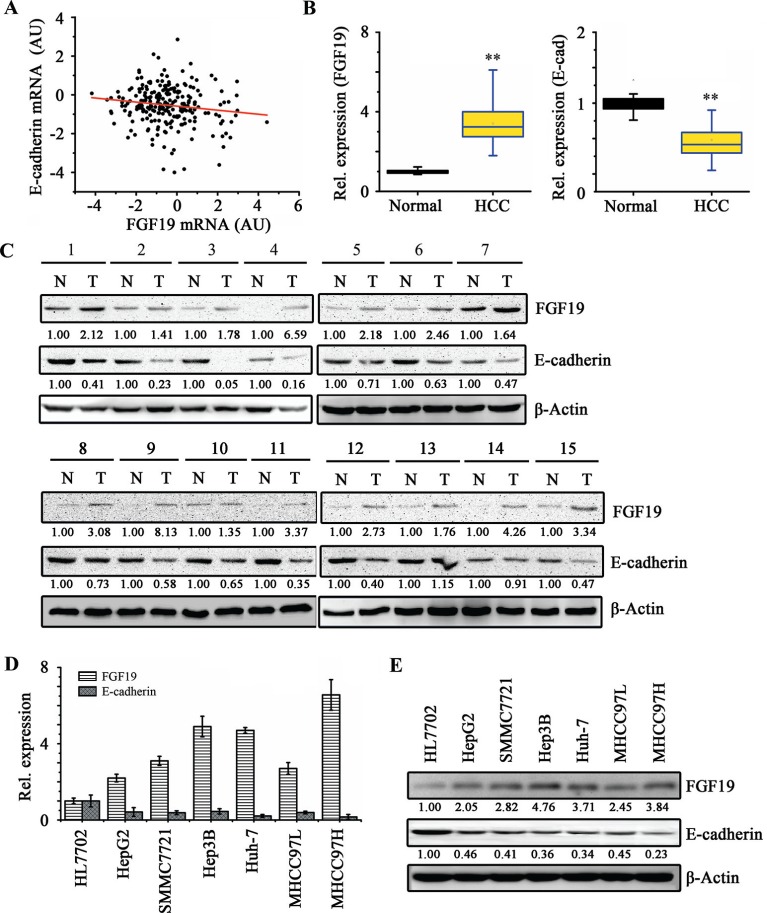
FGF19 and E-cadherin expressions are negatively correlated in HCC tissue samples and cell lines **(A)** Gene expression data of E-cadherin and FGF19 were obtained from GSE5975 dataset (238 HCC tissue samples). The scatter plot of FGF19 and E-cadherin expression showed that they have in negative correlation (Pearson's correlation coefficient = −0.14345, *P* = 0.02691). **(B-C)** Tumor (T) and adjacent non-tumor tissue (N) pairs from patients with HCC were collected and examined for the expression of FGF19 and E-cadherin. RT-qPCR analysis showed a higher average expression of FGF19 and a lower average expression of E-cadherin in HCC tissue samples compared with those in the adjacent normal tissue samples **(B)** Western blot analysis showed that 15 out of 19 (78%) tissue pairs have higher levels of FGF19 and lower levels of E-cadherin in HCC tissue samples as compared to their adjacent normal tissue **(C)** The representative results are shown. RT-qPCR **(D)** and Western blotting **(E)** analysis showed FGF19 and E-cadherin expressions are negatively correlated in HCC cell lines. All error bars in this figure represent S.E.M. (*n* = 3, **P < 0.01).

EMT plays a critical role in metastasis, inducing tumor-associated epithelial cells to obtain mesenchymal features to increase motility [30]. This process is typically mediated by repression of E-cadherin, a key cell adhesion protein implicated as a tumor/invasion suppressor in human cancers [7]. To explore the potential clinical relevance of FGF19/E-cadherin expression in human HCC, we first assessed the association between FGF19 and E-cadherin mRNA levels in HCC clinical samples using microarray data of 238 HCC patients obtained from Gene Expression Omnibus (GEO). Pearson correlation coefficient analysis showed that FGF19 and E-cadherin expressions were in a negative linear association (*R* = −0.14345, *P* = 0.0269; Figure 1A). Subsequently, we collected primary HCC tissues with paired adjacent normal liver tissues for RT-quantitative PCR (RT-qPCR) and Western blotting analyses. Up-regulation of FGF19 and down-regulation of E-cadherin were observed in the HCC samples compared with the paired adjacent normal liver samples (Figure 1B and 1C). We next determined the expression of FGF19 and E-cadherin in a normal liver cell line (HL-7702) and 6 HCC cell lines (HepG2, SMMC7721, Hep3B, Huh-7, MHCC97L and MHCC97H). Consistently, FGF19 expression is elevated in the HCC cells and negatively associated with E-cadherin expression (Figure 1D and 1E). These observations suggest that the balance and interplay between FGF19 and E-cadherin may contribute to progression of HCC.

### FGF19 suppresses E-cadherin expression and promotes EMT and invasion in HCC cells

**Figure 2:**
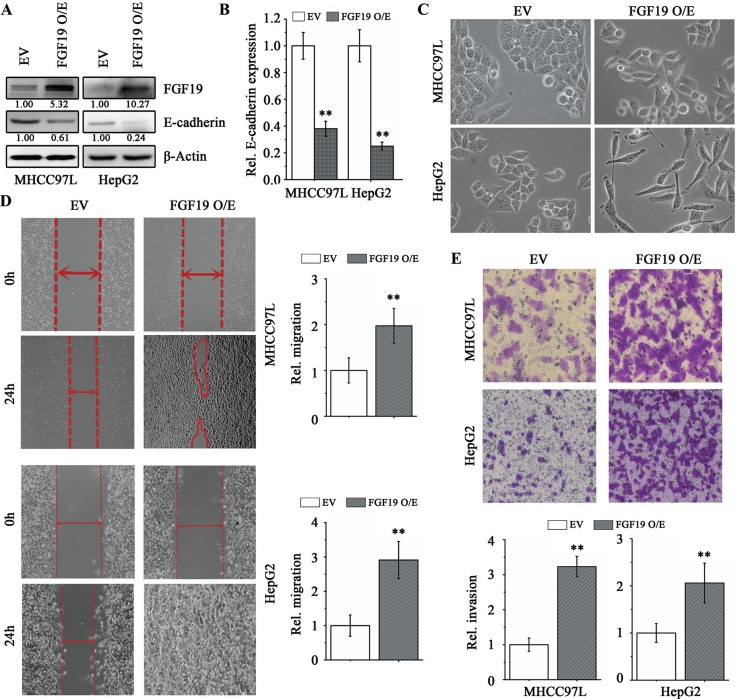
Overexpression of FGF19 in low invasive HCC cells promotes EMT and migration/invasion Western blotting **(A)** and RT-qPCR **(B)** analysis showed ectopic expression of FGF19 (FGF19 O/E) in HepG2 and NHCC97L led to a dramatic decrease in E-cadherin expression compared with the control expressing empty vector (EV). (C-E) Overexpression of FGF19 in low invasive HCC cells facilitates EMT and promotes migration and invasion. Morphologies of control and FGF19 overexpression in MHCC97L and HepG2 cells **(C)** Wound-healing **(D)** and Transwell invasion **(E)** assays for the migration and invasion of control and FGF19 overexpression in MHCC97L and HepG2 cells. All error bars in this figure represent S.E.M. (*n* = 3, **P < 0.01).

To investigate the role of FGF19 in EMT, we overexpressed FGF19 in the epithelial HCC cell lines HepG2 and MHCC97L. There was a remarkable increase in secreted FGF19 levels by the cancer cells when FGF19 was overexpressed (Figure 2A and Supplementary Figure S1). Interestingly, forced expression of FGF19 led to a repression of E-cadherin (Figure 2A and 2B) and elevated expression levels of the mesenchymal-related genes (N-cadherin, Vimentin, Snail1 and Twist) compared with the cells expressing empty vector (Supplementary Figure S2A). Ectopically expressing FGF19 also facilitated transition of epithelial HepG2 and MHCC97L cells to a mesenchymal phenotype (Figure 2C) and enhanced the migration and invasion potential (Figure 2D and 2E). The FGF19-overexpressing cells were maintained at least a month and phenotypic alterations were observed, indicating that FGF19-induced EMT and invasion is stable.

**Figure 3:**
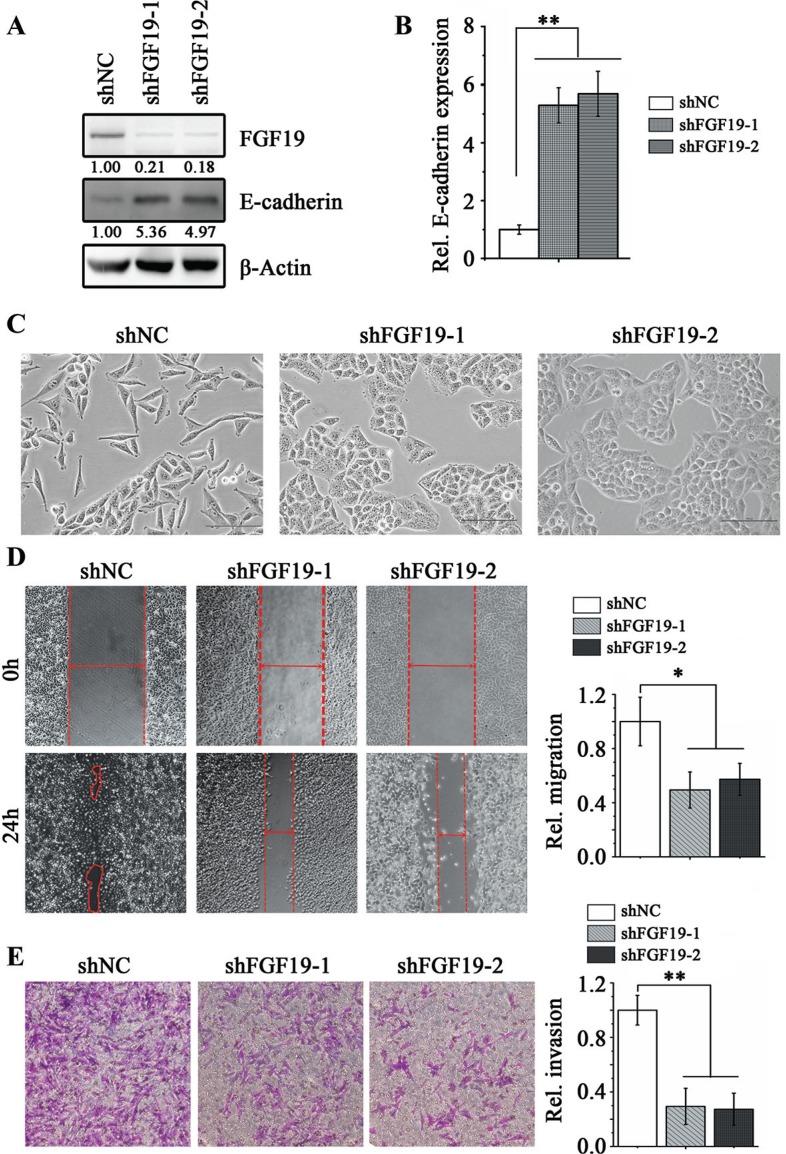
Knockdown of FGF19 in high invasive HCC cells suppresses EMT and migration/invasion Western blotting **(A)** and RT-qPCR **(B)** analysis showed knockdown of FGF19 (shFGF19–1, shFGF19–2) in MHCC97H cells led to a significantly increase in E-cadherin expression compared with the knockdown control (shNC). **(C-E)** Knockdown of FGF19 in high invasive MHCC97H cells suppresses EMT and migration/invasion. Morphologies of control and FGF19 knockdown in MHCC97H cells **(C)** Wound-healing **(D)** and Transwell invasion **(E)** assays for the migration and invasion of control and FGF19 knockdown in MHCC97H cells. All error bars in this figure represent S.E.M. (*n* = 3, *P < 0.05; **P < 0.01).

Elevated expression level of FGF19 and low expression of E-cadherin have been detected in cirrhotic liver [26, 31]. Similar to the observations from HCC cells, overexpression of FGF19 suppressed E-cadherin expression (Figure 3A and 3B) and promoted EMT in HL7702 normal liver cells (Figure 3C), implicating that FGF19-induced EMT may also play a critical role in cirrhotic liver diseases.

Next, we investigated the importance of FGF19 in EMT by lentiviral introducing FGF19 shRNA into mesenchymal-like MHCC97H cells. Knockdown of FGF19 led to dramatically increased epithelial marker E-cadherin expression compared with the knockdown control cells (Figure 3A and 3B). In contrast, loss of FGF19 was associated with decreased expression levels of mesenchymal-related genes (N-cadherin, Vimentin, Snail1 and Twist) in MHCC97H (Supplementary Figure S1B). As expected, FGF19 knockdown diminished EMT traits in MHCC97H cells (Figure 3C) and suppressed cell migration (Figure 3D) and invasion (Figure 3E). Together, these results strongly suggest that FGF19 is a potent inducer of EMT in HCC cells.

### The GSK3β/β-catenin pathway is required for FGF19-induced EMT in HCC cells

**Figure 4:**
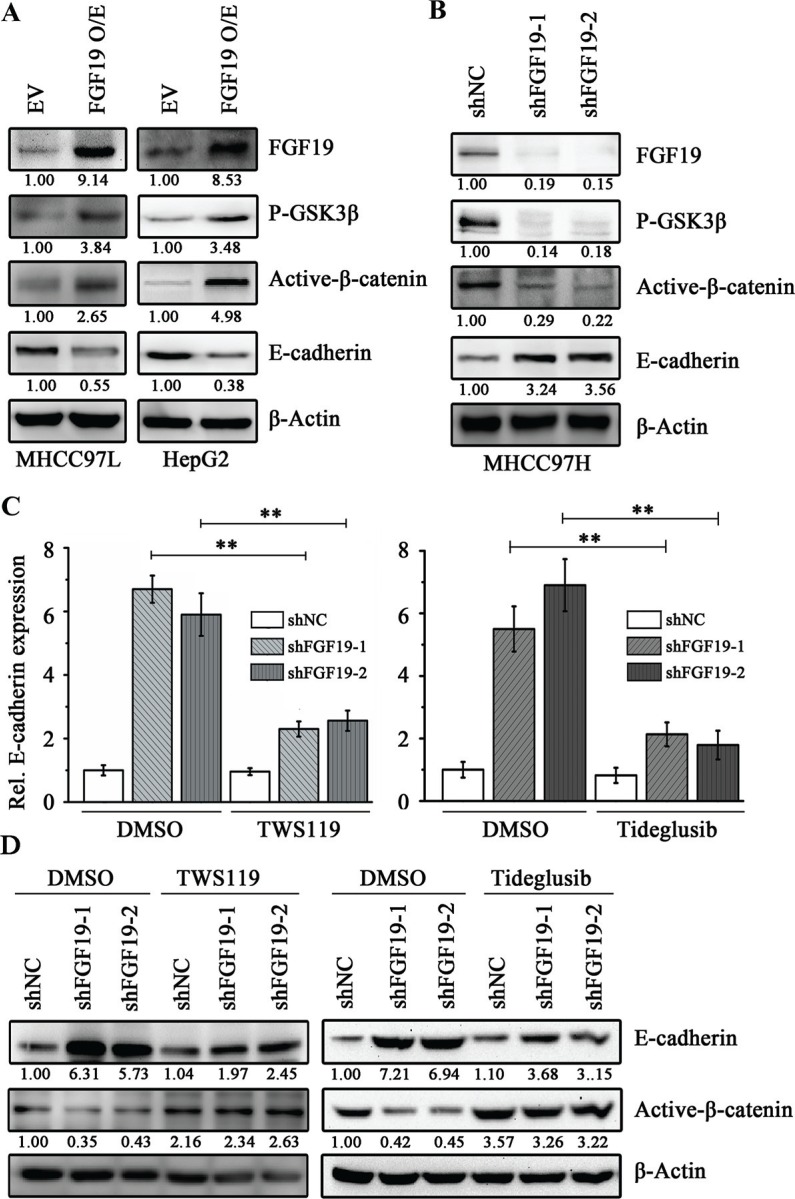
FGF19 induces EMT through GSK3β/β-catenin signaling **(A)** Western blotting analysis showed knockdown of FGF19 in MHCC97H cells inhibited GSK3β phosphorylation and β-catenin activation. **(B)** Western blot analysis showed overexpression of FGF19 enhanced GSK3β phosphorylation and β-catenin activation in MHCC97L and HepG2 cells. RT-qPCR **(C)** and Western blotting **(D)** analysis showed treatment with GSK3β inhibitors TWS119 (10 μM) and Tideglusib (1 μM) for 24 hours suppressed E-cadherin expression in FGF19 knockdown MHCC97H cells. All error bars in this figure represent S.E.M. (*n* = 3, **P < 0.01).

The β-catenin signaling network plays an essential role in EMT of HCC cells through negative regulation of the E-cadherin expression [4]. To determine whether FGF19-induced EMT is functionally associated with the β-catenin signaling pathway, we examined the levels of phospho-GSK3β (inactive GSK3β form) and non-phospho-β-catenin (active β-catenin form) by Western blotting. Overexpression of FGF19 in MHCC97L and HepG2 cells resulted in a significant up-regulation of phospho-GSK3β and active β-catenin (Figure 4A). In contrast, knockdown of FGF19 in MHCC97H cells reduced phospho-GSK3β and downstream active β-catenin levels (Figure 4B).

Aberrant GSK3β activity has been linked with several human diseases including cancer [12]. Inhibiting GSK3β activity via pharmacological intervention has become an important strategy for inhibition of its function [32]. To determine whether the GSK3β/β-catenin signaling plays an essential role in FGF19-induced EMT in HCC cells, we inactivated GSK3β function using a GSK3β inhibitor TWS119, which leads to reduced β-catenin phosphorylation and induces nuclear translocation of β-catenin [33]. Interestingly, after TWS119 treatment, a remarkable decrease in E-cadherin expression was observed following increased levels of active β-catenin in FGF19 knockdown MHCC97H cells (Figure 4C and 4D). Consistently, the epithelial-like morphology was reverted back to mesenchymal-like in treatment with TWS119 (Data not shown). The similar results were found in FGF19 knockdown MHCC97H cells when treated by another GSK3β inhibitor Tideglusib (Figure 4C and 4D). However, treated of FGF19-overexpressing MHCC97L cells with other pathway inhibitors (such as BEZ235 and GW5074) cannot rescue the reduction of E-cadherin expression (Supplementary Figure S4). These observations demonstrate that GSK3β/β-catenin pathway is required for FGF19-promoting EMT in HCC cells.

### FGFR4 activation is required for FGF19-induced EMT in HCC cells

FGFR4 is the predominant FGFR isoform present in human hepatocytes and both of FGF19 and FGFR4 are highly expressed in primary HCC [34]. It has been shown that FGFR4 has a non-substitutable role in modulating FGF19 activity to liver such as tumor growth and development [35, 36]. Our data revealed that FGFR4 overexpression repressed E-cadherin expression by activation of GSK3β/β-catenin pathway in MHCC97L cells (Supplementary Figure S5).

**Figure 5:**
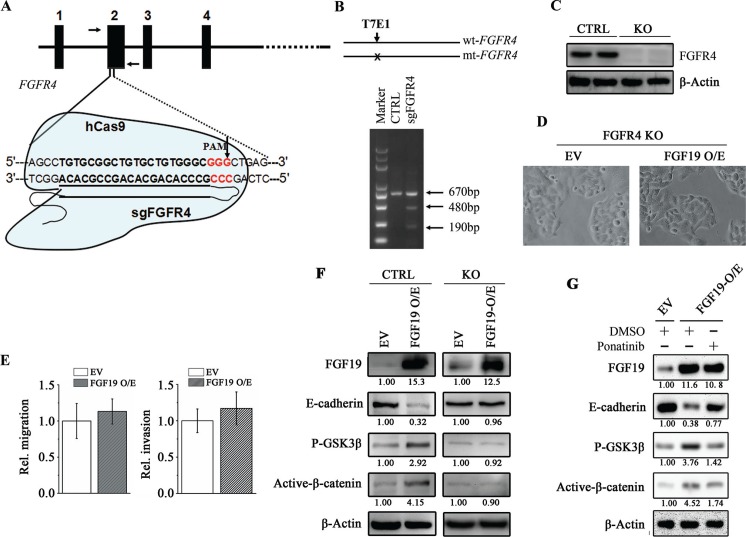
FGFR4 plays a critical role in FGF19-induced EMT **(A)** Diagram depicts that the nuclease hCas9 recruited by a single guided RNA (sgRNA) specifically recognizing a region spanning the FGFR4 codon (sgFGFR4) cleaves the FGFR4 gene. The vertical arrow showed the potential cleavage site. **(B)** The T7E1 nuclease cleaves the mismatch site of a hetero duplex DNA (670 bp) formed by the wild-type and mutated FGFR4 gene fragments amplified by PCR using the primers indicated by arrows in a, which generates two cleaved products (480 and 190 bp). **(C)** Western blotting analysis showed no FGFR4 was detected in the sgFGFR4-targeting (FGFR4-KO) MHCC97L cells. (D, E) FGF19 cannot induce cell EMT and promote migration/invasion in the absent of FGFR4 expression. Morphologies of control and FGF19-overexpressing FGFR4-KO cells **(D)** Wound-healing and Transwell invasion assays for the migration and invasion potential of control and FGF19-overexpressing FGFR4-KO cells **(E, F)** Western blot analysis showed FGF19 overexpression cannot increase GSK3β phosphorylation and β-catenin activation in the FGFR4-KO cells. **(G)** Western blot analysis showed increased GSK3β phosphorylation and β-catenin activation by FGF19 overexpression was reduced when treated with FGFR4 inhibitor Ponatinib for 24 hours.

To evaluate the role of FGFR4 in FGF19-induced EMT in HCC cells, we successfully generated genomic FGFR4 knockout MHCC97L cells by CRISPR-Cas9 system (Figure 5A–5C). The well-designed single guided RNA (sgRNA) specifically binds to the region on FGFR4 exon2 and recruits a nuclease Cas9 to cleave DNA (Figure 5A). The T7E1-based mutagenesis detection assays showed the clones isolated carries at least one mutant FGFR4 allele (Figure 5B). Western blotting analysis further showed that no FGFR4 protein was detected in these monoclonal cells (Figure 5C). We then used FGFR4 knockout cells to determine the FGFR4 function in FGF19-induced EMT. As described above, overexpression of FGF19 in the epithelial HCC cells promotes cell EMT, migration and invasion (Figure 2). However, when FGFR4 was knocked out, FGF19 overexpression cannot change the cell epithelial phenotype and the capacity of cell migration and invasion (Figure 5D and 5E). Moreover, FGF19 overexpression in FGFR4 depletion cells cannot inhibit E-cadherin expression and failed to induce the activation of GSK3β/β-catenin pathway compared with the control cells (Figure 5F). Ponatinib, an inhibitor for the *in vitro* kinase activity of FGFR4, is shown to enhance the E-cadherin expression in MHCC97H (Supplementary Figure S6). Ponatinib treatment prevented the reduction of E-cadherin levels induced by FGF19 overexpression through blocking the activation of GSK3β/β-catenin pathway (Figure 5G). These results thus strongly demonstrate that FGFR4 is required for FGF19-induced EMT in HCC cells.

## DISCUSSION

FGF19 is contained within a focal amplification on chromosome 11q13.3 in the patients with HCC [37], which may be a key driver in certain forms of HCC. In HCC, increasing evidence indicates a central role of EMT which might occur at the leading edge of tumor cells under the particular control of extrinsic factors derived from the tumor microenvironment [38]. FGF19 has been reported to be secreted from multiple cell types within the tumor microenvironment and functioned as both autocrine and paracrine factors on tumor cells and stromal cells [39, 40]. Mechanistic study shows tumor-associated fibroblasts (TAF) -derived FGF19 is required for TAF-induced FGFR4/Wnt activation and governs colorectal cancer cell metastasis *in vivo* [39]. Using gain- and loss-of-function approaches instead of recombinant FGF19 treatment, we investigated the FGF19 function in the stable FGF19 overexpressing and knockdown HCC cells. Overexpression of FGF19 can increase the FGF19 protein levels in the supernatant, suggesting that FGF19 can be secreted by HCC cells and then activates its specific receptor FGFR4.

**Figure 6:**
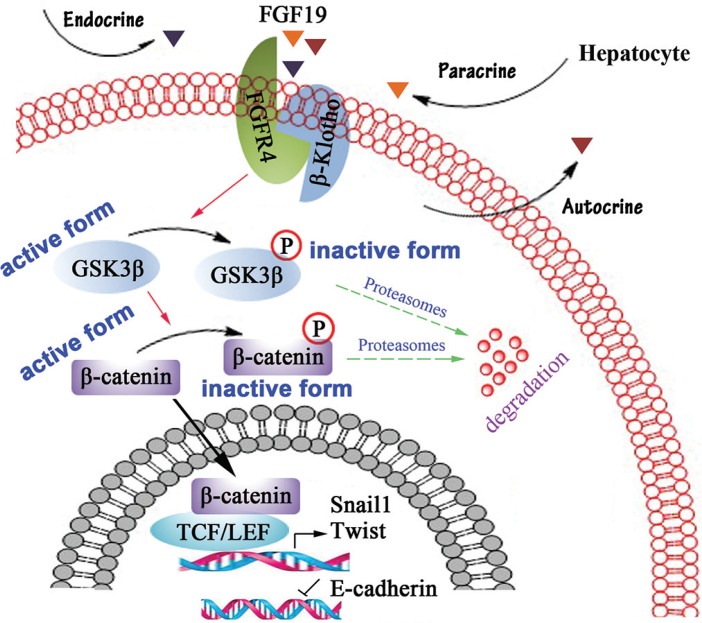
Schematic representation of FGF19/FGFR4 signaling pathway involves in EMT process in HCC cells In HCC cells, high concentration of FGF19 from autocrine, paracrine and endocrine secretion constitutively activates FGFR4, resulting in phosphorylating GSK3β at S9, which in favor of increased active β-catenin in nuclear. Nuclear β-catenin represses E-cadherin expression to promote EMT through enhancing the expression of Snail1 and Twist.

It has been shown that activation of FGFR4 facilitates β-catenin nucleus translocation through phosphorylating GSK3β at S9 [28]. Snail1 and Twist are well-known β-catenin target genes and E-cadherin repressors [41, 42]. Overexpression of Snail1 or/and Twist in Huh-7 induces EMT, invasion and metastasis, whereas knockdown of them in Mahlavu suppresses these phenotype [8]. Our present data implicate a critical signaling that controls the EMT process. In HCC cells, high concentration of FGF19 from autocrine, paracrine and endocrine secretion constitutively activates FGFR4, resulting in phosphorylation of GSK3β, which in favor of increased active β-catenin. Active β-catenin then translocates into the nucleus and initiates expression of Snail1 and Twist, which subsequently represses E-cadherin expression and facilitates EMT in HCC cells (Figure 6).

FGFR4 are most highly expressed in liver and its expression was found to be significantly elevated in about one-third of HCC patients [34]. To test the hypothesis that function of FGFR4 is responsible for the FGF19-induced EMT in HCC, we generated FGFR4 knockout MHCC97L cells and overexpressed FGF19 in this cell line. No changes were observed in cell shapes and metastasis when overexpression of FGF19, indicating that FGFR4 largely contributes to FGF19-induced EMT. FGF19 drives HCC development through the regulation of multiple signaling pathways. To elucidate the mechanism involved in FGF19-induced EMT, we treated FGF19 modulated cells with different pathway inhibitors. Only GSK3β inhibitors (TWS119 and Tideglusib) can prevent the change of E-cadherin expression and cell morphology following FGF19 gene modification in HCC cells. These observations suggest that the effect of FGF19 on EMT is likely induced by activating the GSK3β/β-catenin pathway, which is dependent on FGFR4 activation.

Suppression of EMT is crucial for improving treatment of hepatocellular carcinoma (HCC). Different from other cancer types, blocking of FGFR4 has side effects for HCC patients. Treatment with FGFR4 inhibitors increases a risk of bile acid metabolism disorder [43]. It has been reported that the anti-FGF19 antibody 1A6 represses β-catenin activation in colorectal cancer xenograft models [26] and an engineered FGF19 (M70) inhibits FGF19-dependent tumorigenesis but retains the ability to maintain bile acid homeostasis. Our findings suggest that FGF19-FGFR4 signaling pathway promotes EMT in HCC, which makes the pathway an interesting, emerging molecular target for potential therapeutic intervention. Given that there are currently no genetically-targeted therapies for HCC, inactivation of FGF19 without influencing liver metabolism or selective disruption of FGF19-FGFR4 interaction may be beneficial in treatment for metastatic HCC. Thus, we believe our results represent a potential therapeutic advance to improve overall survival of HCC patients.

## MATERIALS AND METHODS

### Human HCC specimens

All samples of primary HCC tissues with paired adjacent normal liver tissues for RT-qPCR and Western blotting analyses were from Third Affiliated Hospital of Third Military Medical University (Chongqing, China). Informed consent was given by all of the patients and all samples were histologically confirmed before analysis.

### Cell cultures and standard assays

HL7702, HepG2, Huh-7, MHCC97L, MHCC97H, Hep3B and SMMC7721 cells were purchased from the Cell Bank of Type Culture Collection of Chinese Academy of Sciences (Shanghai, China) and maintained according to the supplier's instructions.

### Wound-healing and transwell invasion assays

For wound-healing migration assays, a single scratch wound was created by dragging a 100 μl plastic pipette tip across the cell surface. The area of a defined region within the scratch was measured using ImageJ software. The extent to which the wound had closed over 24 h was calculated and expressed as a percentage of the difference between times 0 and 24 h. For invasion assays, Transwells (BD biosciences, San Jose, CA) with 8-μm pore size filters covered with matrigel were inserted into 24-well plates. The cells were serum-starved overnight and then added in the upper chamber (2.5 × 10^4^ cells per insert) and the complete culture medium supplemented with 10% FBS was used as a chemoattractant in the lower chamber. After 24 h of incubation, non-invading cells that remained on the upper surface of the filter were removed, and the cells that had passed through the filter and attached to the bottom of the membrane were fixed in methanol and stained with 0.2% Crystal violet. Numbers of the invasive cells in seven randomly selected fields from triplicate chambers were counted in each experiment under a phase-contrast microscope.

### Antibodies, inhibitors and constructs

FGF19 and β-Actin antibodies were purchased from Abcam (Cambridge, MA) and Sigma (St Louis, MO), respectively. Antibodies against FGFR4, phospho-GSK3β (Ser9), E-cadherin and non-phospho β-Catenin (active) were from Cell Signaling (Beverly, MA). GSK3β inhibitors TWS119 and Tideglusib, PI3K inhibitors BEZ235 and LY294002, p38 inhibitor GW5074, JNK inhibitor SP600125 and MEK inhibitors U0126 and PD98059 were obtained from Selleckchem (Houston, TX, USA). The full-length of human FGF19 and FGFR4 was PCR amplified from Huh-7 cells. The following primers were used: 5′-CCC AAG CTT GGG ATG CGG AGC GGG TGT GTG GTG GTC CAC-3′ (FGF19-forward); 5′-CCG CTC GAG CGG CTA CTT CTC AAA GCT GGG ACT CCT CAC-3′ (FGF19-reverse); 5′-AGT AAG CTT ATG CGG CTG CTG CTG GCC CT-3′ (FGFR4-forward) and 5′-GAT CTC GAG TCA TGT CTG CAC CCC AGA CC-3′ (FGFR4-reverse). The gene FGF19 was cloned into pcDNA3.1 (+) expression vector (Invitrogen, USA) using Hind III and XhoI sites (FGF19 O/E). The gene FGFR4 was cloned into pcDNA3.1 (+) vector using KpnI and XhoI (FGFR4 O/E). Lentiviral vectors harboring shRNAs targeting FGF19 were designed and obtained from GeneCopoeia (Rockville, MD). The stable FGF19 knockdown MHCC97H and Hep3B cells were generated using Lenti-Pac™ FIV Expression Packaging Kit (GeneCopoeia) according to the manufacturer's instructions. Non-target shRNA (shNC) was used as a negative control in this study and the knockdown effect was confirmed by Western blotting. All the plasmids used in this study were verified by sequencing.

### CRISPR-Cas9 targeted deletion of FGFR4

To knock out FGFR4 in MHCC97L cells, we designed sgRNA sequences (Forward 5′-CAC CGT GTG CGT CTG TGC TGT GGG C-3′; Reverse: 5′-AAA CGC CCA CAG CAC AGA CGC ACA C-3′) for human FGFR4 gene and cloned the targeting sequences into the lentiCRISPR v2 vector that was obtained from Feng Zhang (Addgene plasmid # 52961). Lentivirus for FGFR4 sgRNA and vector control were generated in 293FT cells by standard methods using amphotropic packaging vector. MHCC97L cells were then infected with lentivirus for 48 hours and selected with puromycin for 10 days. FGFR4 mRNA and protein levels were analyzed 2 weeks after infection. The FGFR4 deletion of individual clones was further verified by sequencing.

### RT-qPCR and western blotting analysis

Total RNA was extracted from cells and tissues using Trizol (Life technologies, NY) according to the manufacturer's instructions. Upon isolating RNA, DNase I was treated to eliminate contaminating genomic DNA. RevertAid First Strand cDNA Synthesis Kit (Life technologies, NY) was used for cDNA synthesis. All results were quantified by qPCR performed using GoTaq qPCR Master Mix (Promega, WI) on a BioRad CFX96 (Bio-Rad, CA). Gene expression levels were analyzed using the delta Ct method and normalized against β-actin. The gene-specific primers used in RT-qPCR were listed in Table 1.

**Table 1:** Primers for RT-qPCR in this study

Gene name	Forward (5′–3′)	Reverse (5′–3′)
FGF19	GGAGATCCGCCCAGATGGCTAC	GGCCTCCAGTCCGGTGACAAGC
FGFR4	GGCCTCCAGTCCGGTGACAAGC	CCACAGCGTTCTCTACCAGG
E-cadherin	TGAAGGTGACAGAGCCTCTGGAT	TGAAGGTGACAGAGCCTCTGGAT
N-cadherin	CACTGCTCAGGACCCAGAT	TAAGCCGAGTGATGGTCC
Vimentin	CACGCCTGGCACTGGTACTTCT	CACGCCTGGCACTGGTACTTCT
Snail1	ATCGGAAGCCTAACTACAGCGA	CACGCCTGGCACTGGTACTTCT
Twist	GGAGTCCGCAGTCTTACGAG	GGAGTCCGCAGTCTTACGAG
β-actin	CATGTACGTTGCTATCCAGGC	CTCCTTAATGTCACGCACGAT

For human tissues, samples were harvested and lysed in RIPA buffer using a dounce homogenizer, followed by sonication and incubation on ice for 15 min. Pellets were spun down and the supernatant was collected. For cells, whole lysates were prepared by direct lysis in RIPA buffer. Proteins were quantified using Pierce BCA Protein Assay Kit (Life technologies, NY) and 40 μg per well were loaded. Samples were then separated by SDS-PAGE electrophoresis and transferred to nitrocellulose for detection using antibodies.

The abundance of the proteins in western blot assays was determined by densitometry using Quantity One 1D Analysis Software v4.6 (Bio-Rad, CA).

### Detection of FGF19 secretion in culture supernatant by ELISA assays

FGF19 levels in supernatants were measured using the Human FGF19 Quantikine^®^ ELISA kit (R&D Systems, MN) according to the manufacturer's protocols. Secreted FGF19 levels were read at 450 nm within 30 minutes.

### Statistical analysis

Statistical analyses were performed using SPSS16.0 for Windows. Data were presented as mean ± S.E.M of at least three independent experiments. Statistical analysis was performed using Student's *t* test and one-way ANOVA. The Pearson's correlation coefficient was used to analyze the correlation between E-cadherin and FGF19 gene expression that was obtained from GEO database. A *P*-value of 0.05 or less was considered to be significant.

## SUPPLEMENTARY MATERIAL FIGURES


