# Supplementary material for: FGF19 promotes epithelial-mesenchymal transition in hepatocellular carcinoma cells by modulating the GSK3β/β- catenin signaling cascade via FGFR4 activation
Source: Oncotarget. 2015 Oct 20;7(12):13575–86. doi: 10.18632/oncotarget.6185 (PMC4924662; doi:10.18632/oncotarget.6185)
Supplement: Supplementary file 1 [file oncotarget-07-13575-s001.pdf]

FGF19 promotes epithelial-mesenchymal transition in hepatocellular carcinoma cells by modulating the GSK3β/β-catenin signaling cascade via FGFR4 activation

Supplementary Materials

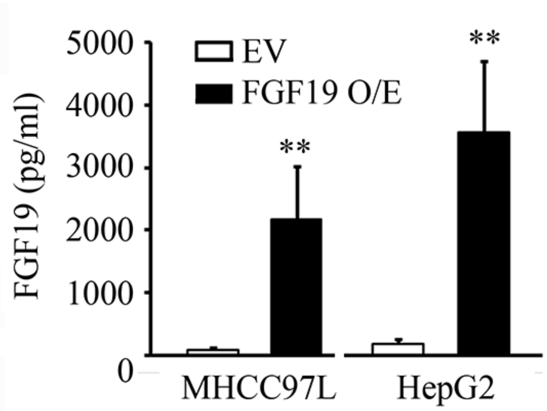

Supplementary Figure S1: Overexpression of FGF19 in MHCC97L and HepG2 cell led to a significant increase in secreted FGF19 proteins in the supernatant ($n = 6$, $**P < 0.01$).

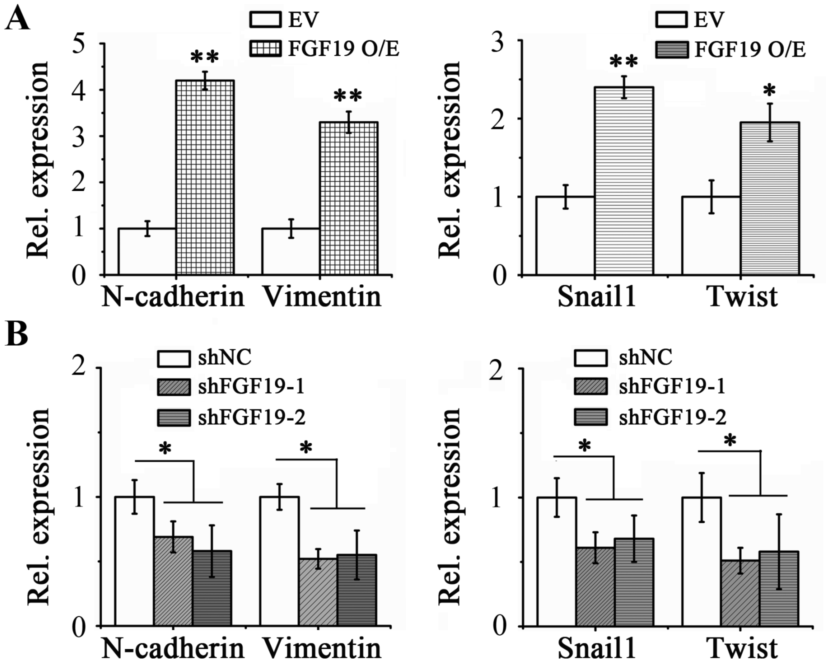

Supplementary Figure S2: **A.** RT-qPCR analysis showed ectopic expression of FGF19 (FGF19 O/E) in MHCC97L cells increased the expression levels of N-cadherin, Vimentin, Snail1 and Twist. **B.** RT-qPCR analysis showed knockdown of FGF19 (shFGF19-1 and shFGF19-2) in MHCC97H cells led to a decrease in the expression of N-cadherin, Vimentin, Snail1 and Twist. All error bars in this figure represent S.E.M. ($n = 3$, $*P < 0.05$; $**P < 0.01$).

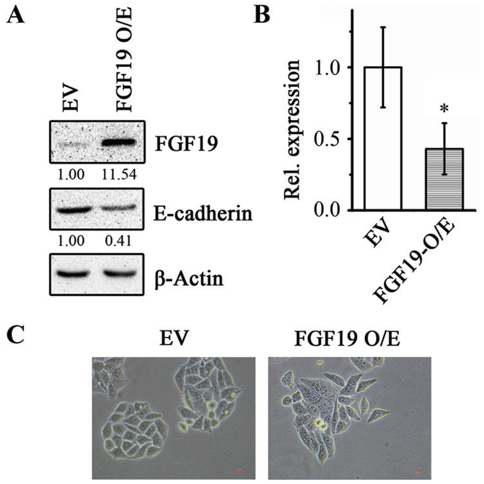

Supplementary Figure S3: **A–B.** Western blotting and RT-qPCR analysis showed overexpression of FGF19 (FGF19 O/E) in liver normal HL7702 cells led to a suppression of E-cadherin expression compared with the control cells expressing empty vector (EV). **C.** Overexpression of FGF19 facilitats epithelial HL7702 cells to mesenchymal morphology. All error bars in this figure represent S.E.M. ($n = 3$, $*P < 0.05$).

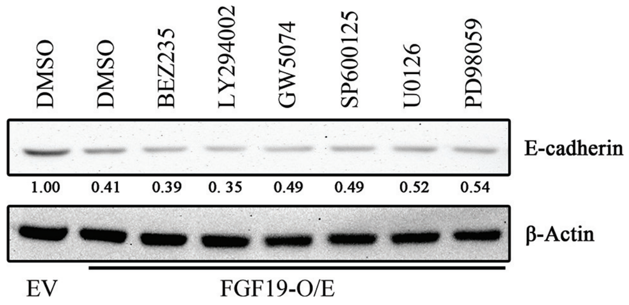

Supplementary Figure S4: Western blotting analysis showed treatment with PI3K inhibitors BEZ235(20 nM) and LY294002 (10 μM), p38 inhibitor GW5074 (10 μM), JNK inhibitor SP600125 (10 μM) and MEK inhibitors U0126 (1 μM) and PD98059(10 μM) for 24 hrs cannot prevent the reduction of E-cadherin expression in FGF19 overexpressing MHCC97L cells.

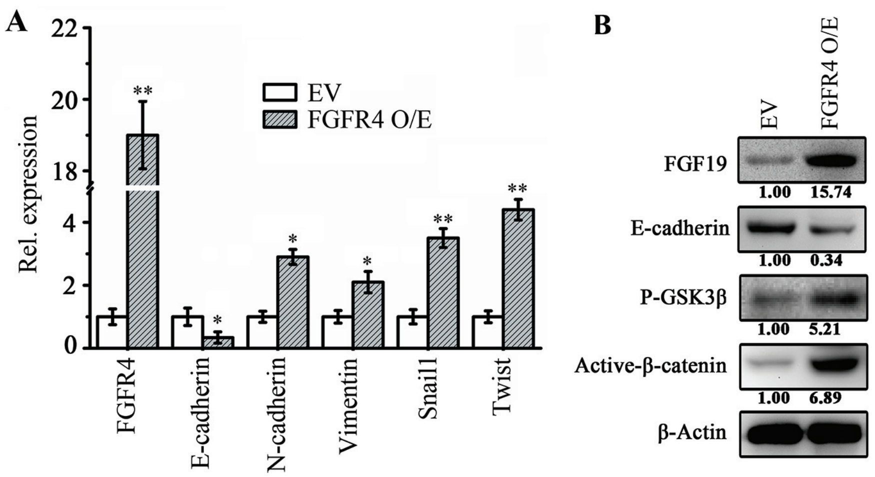

Supplementary Figure S5: **A.** RT-qPCR analysis showed overexpression of FGFR4 (FGFR4 O/E) in MHCC97L cells led to a decrease in E-cadherin expression and an increase in the expression of N-cadherin, Vimentin, Snail1 and Twist. **B.** Western blotting analysis showed overexpression of FGFR4 (FGFR4 O/E) in MHCC97L cells activated GSK3β/ β-catenin axis. All error bars in this figure represent S.E.M. ($n = 3$, $^*P < 0.05$; $^{**}P < 0.01$).

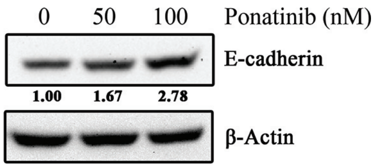

Supplementary Figure S6: Western blotting analysis showed treatment with FGFR4 inhibitor ponatinib for 24 hrs enhanced the expression of E-cadherin in MHCC97H cells.
